# Complete Genome Sequencing and Comparative Genomic Analysis of the Thermotolerant Acetic Acid Bacterium, *Acetobacter pasteurianus* SKU1108, Provide a New Insight into Thermotolerance

**DOI:** 10.1264/jsme2.ME16023

**Published:** 2016-09-24

**Authors:** Minenosuke Matsutani, Hideki Hirakawa, Eri Hiraoka, Gunjana Theeragool, Toshiharu Yakushi, Kazunobu Matsushita

**Affiliations:** 1 Department of Biological Chemistry, Faculty of Agriculture, Yamaguchi UniversityYamaguchi 753–8515Japan; 2 Kazusa DNA Research InstituteKazusa-kamatari, Kisarazu, Chiba 292–0818Japan; 3 Department of Microbiology, Faculty of Science, Kasetsart UniversityBangkok 10900Thailand; 4 Research Center for Thermotolerant Microbial Resources, Yamaguchi UniversityYamaguchi 753–8515Japan

**Keywords:** acetic acid bacterium, thermotolerant strain, complete genome sequence

## Abstract

*Acetobacter pasteurianus* SKU1108 is a typical thermotolerant acetic acid bacterium. In this study, the complete genome sequence of the SKU1108 strain was elucidated, and information on genomic modifications due to the thermal adaptation of SKU1108 was updated. In order to obtain a clearer understanding of the genetic background responsible for thermotolerance, the SKU1108 genome was compared with those of two closely related complete genome strains, thermotolerant *A. pasteurianus* 386B and mesophilic *A. pasteurianus* NBRC 3283. All 24 “thermotolerant genes” required for growth at higher temperatures in the thermotolerant *Acetobacter tropicalis* SKU1100 strain were conserved in all three strains. However, these thermotolerant genes accumulated amino acid mutations. Some biased mutations, particularly those that occurred in xanthine dehydrogenase XdhA, may be related to thermotolerance. By aligning whole genome sequences, we identified ten SKU1108 strain-specific regions, three of which were conserved in the genomes of the two thermotolerant *A. pasteurianus* strains. One of the regions contained a unique paralog of the thermotolerant gene *xdhA*, which may also be responsible for conferring thermotolerance. Thus, comparative genomics of complete genome sequences may provide novel insights into the phenotypes of these thermotolerant strains.

Strictly aerobic acetic acid bacteria (AAB) are classified as a sub-group of the *Acetobacteraceae* family in the class *Alphaproteobacteria*. AAB strongly oxidize various sugars, alcohols, and sugar alcohols. Vinegar is industrially produced by oxidative fermentation using AAB, particularly those in *Acetobacter* and *Komagataeibacter* (37). In ethanol-containing medium, the bacterial strains of these two genera exhibit three growth phases; an ethanol oxidation phase, acetic acid resistance phase, and acetate over oxidation phase (14, 19). In the ethanol oxidation phase, cells oxidize ethanol to acetic acid via acetoaldehyde with membrane-bound alcohol and aldehyde dehydrogenases linked to the respiratory chain (20). In the acetic acid resistance phase, cells resist autoproduced acetic acid using several mechanisms without assimilating it. In the overoxidation phase, cells assimilate acetate by oxidizing it to CO_2_ via the TCA cycle (14, 19).

The *Acetobacter pasteurianus* SKU1108, isolated from fruits in Thailand, has been shown to efficiently perform acetic acid fermentation at higher temperatures than the required temperature range for other *A. pasteurianus* strains, such as NBRC 3283 and IFO 3191 (=NBRC 3191) (21, 28). We previously reported the complete genome sequence of the mesophilic strain, *A. pasteurianus* NBRC 3283 (2). Furthermore, the complete genome sequence of the thermotolerant *A. pasteurianus* 386B has recently been published (13). Therefore, in order to identify the genes responsible for the specific thermotolerance phenotype, we elucidated the complete genome sequence of the *A. pasteurianus* SKU1108. Comparisons of this sequence with those of other thermotolerant and mesophilic strains enabled us to identify the genomic regions conserved in thermotolerant bacteria.

## Materials and Methods

### Genomic DNA and library preparation

Genomic DNA from SKU1108 for genome sequencing was prepared as previously reported (23). Genomic DNA (~20 μg) was purified with the AMPure Xp Kit (Beckman Coulter, Beverly, MA, USA). DNA was sheared using a Covaris g-TUBE (Covaris, Woburn, MA, USA) following the manufacturer’s recommendations for 20-kb fragments. A sequencing library was prepared using the SMRTbell Template Prep Kit 1.0 (Pacific Biosciences of California, Menlo Park, CA, USA). Libraries for 2 SMRT cells (~10 μg) were constructed using two different methods. Small library fragments were removed using BluePippin from one SMRT cell (Sage Science, Beverly, MA, USA) with a 10-kb cut-off. In the other cell, small library fragments were not removed.

### DNA sequencing and assembly

Genome sequencing was performed by Takara Bio using PacBio RSII single-molecule real-time (SMRT) sequencing technology (Pacific Biosciences of California, Menlo Park, CA, USA). The sequence data obtained from 2 SMRT cells were used for the subsequent sequence assembly. Sequencing reads were assembled using Hierarchical Genome Assembly Process 3 (HGAP3) in PacBio SMRT portal version 2.3.0. Three large contigs were assembled with a mean coverage of 387-fold. The assembly was corrected with the Quiver consensus algorithm to obtain a high-accuracy genome assembly (7). The overlap sequences of each contig end were manually edited. Previously reported Illumina sequence reads of the SKU1108 strain were mapped onto these three contigs using Bowtie 2 (18, 22). Unmapped reads were collected and a *de novo* assembly was performed using SPAdes 3.0.0 (3). The resulting assembly revealed two additional small plasmids: plasmids 3 and 4.

### Genome annotation

The gene detection and genome annotation of the chromosome and four plasmid sequences were performed using the auto-annotation package Prokka (29). Protein-coding sequences (CDS) of the complete genome sequence were predicted using Prodigal 2.62 (11, 12). ARAGORN 1.26 and Barrnap 0.4 were used to predict tRNA and rRNA regions, respectively (16). The functional assignments of the predicted CDSs were based on a BLASTP homology search against the previously reported *Acetobacter pasteurianus* genome and the NCBI non-redundant (NR) database (1). The positions of the start codon were manually checked and edited using the commercial genome sequence editing software, *in silico* Molecular Cloning v5.3.75 (In Silico Biology, Kanagawa, Japan). All signal peptide genes encoded by the SKU1108 genome were predicted by SignalP 4.1 (26). Clustered-regularly interspaced short palindromic repeats (CRISPRs) in the genome sequence were revealed by MinCED 0.16 (4).

### Whole genome alignment and genome map

The genomic sequences of *A. pasteurianus* 386B (HF677570–HF677572) and *A. pasteurianus* IFO 3283-01 (=NBRC 3283) (AP011121–AP011127) were downloaded from the NCBI FTP website at ftp.ncbi.nlm.nih.gov. The chromosome sequences of the two *A. pasteurianus* strains, 386B and NBRC 3283, were independently aligned against that of the strain SKU1108 using NUCmer (15). Unaligned regions were manually assigned as specific regions 1 to 10. The genes located at these specific regions were identified and listed. We generated graphic illustrations of genome alignments using CGView (33). Graphical alignments were generated using the progressiveMauve program with default parameters (8).

### Identification of genes associated with thermotolerance

The previously reported mutated sites in the genomes of the TI and TH-3 strains associated with thermotolerance were re-confirmed using a previously reported method (22). The 24 thermotolerant genes conserved in *A. tropicalis* NBRC 101654 (=SKU1100) and their homologous sequences were also searched for in the three *A. pasteurianus* complete genomes by BLASTP with an E-value cut-off of 10^−10^ and sequence overlap (query and subject) ≥70% (1, 30).

### Phylogenetic tree construction

A BLASTP search against all proteins encoded by 13 *Acetobacter* and 1 *Gluconacetobacter diazotrophicus* Pal 5 genomes was performed using the amino acid sequences of AarC and AarC1 in *A. pasteurianus* SKU1108 as a query. The resulting hits were aligned using MUSCLE v.3.8.31 at the amino acid sequence level (9, 10). Poorly aligned regions were removed using GBLOCKS version 0.91b (6, 34). A phylogenetic tree was constructed using the PROTGAMMAWAG model in RAxML 8.0.14 with 1,000 bootstrap replicates and visualized with the MEGA 6.1 package (31, 32, 35).

### Sequence data deposition

The *A. pasteurianus* SKU1108 (=NBRC 101655) genome sequence was deposited in DDBJ/EMBL/GenBank under the accession numbers AP014881 to AP014885. The versions described here are the first versions. The BioProject ID is PRJDA65545.

## Results and Discussion

### General genome features

The genomic DNA of *A. pasteurianus* SKU1108 consisted of a 2,902,389-bp circular chromosome and four plasmids: plasmid 1 (187,193 bp), plasmid 2 (6,331 bp), plasmid 3 (2,799 bp), and plasmid 4 (2,278 bp), with a GC content of 52.75% (Table 1). In total, 2,662 and 214 CDSs were identified in the chromosome and plasmids, respectively. Putative functions were assigned to 2,002 genes. Signal sequences were searched against all CDSs, and detected in 252 cases (data not shown). In total, 56 tRNAs, 1 transfer-messenger RNA (tmRNA), and 5 sets of ribosomal RNA operons (*rrn*) were predicted in the chromosome sequence (Table 1).

The genomes of *A. pasteurianus* are known to contain numerous genes encoding membrane-bound oxidoreductases. In the SKU1108 genome, we revealed genes that code for the membrane-bound PQQ-dependent alcohol dehydrogenase (ADH), *adhAB* operon (locus_tag: APT_00084–APT_00083) and its subunit III, *adhS* (APT_00654), and other PQQ enzymes, such as the membrane-bound glucose dehydrogenase (APT_00581), PQQ-dependent dehydrogenase 1 (APT_02219), and PQQ-dependent dehydrogenase 4 (APT_01465). In addition, genes encoding two membrane-bound aldehyde dehydrogenase operons (APT_00973–APT_00975 and APT_00426–APT_00428) were identified. These genes were also conserved in complete genomes of the strains 386B and NBRC 3283. The respiratory chains of *Acetobacter* species are known to play crucial roles in energy metabolism (24, 27). Therefore, the gene repertoires of respiratory chains were investigated. The cytochrome *ba_3_* ubiquinol oxidase *cyaBACD* operon (APT_00087–APT_00090), cytochrome *bd* ubiquinol oxidase *cydBACD* operon (APT_01543–APT_01546), cyanide-insensitive ubiquinol oxidase (CIO) *cioBA* operon (APT_02213–APT_02214), type I NADH-quinone oxidoreductase, *nuoA-nuoN* operon (APT_01737–APT_01725) and *nuoM* (APT_00690), two type II NADH dehydrogenase *ndh* (APT_00547 and APT_02111), heme A synthase *ctaA* (APT_00075), heme O synthase *ctaB* (APT_01774), and cytochrome *c* oxidase subunit *ctaD* (APT_01775) were conserved in the complete genomes of the strains SKU1108, 386B, and NBRC 3283. In contrast, the cytochrome *b* subunit *petB* (APT_01924) of the ubiquinol-cytochrome *c* reductase (*bc_1_* complex) operon (APT_01924-APT_01926) was disrupted with a stop codon insertion around the start codon site in SKU1108, suggesting that this *bc_1_* complex is not functional in SKU1108. However, a paralog sequence of this gene was also found in the SKU1108 genome (APT_00754). Therefore, a combination of these remaining subunits may construct an active *bc_1_* complex.

### Whole genome level comparison in complete genome sequences of three closely related strains

Illeghems and co-workers elucidated the complete genome sequence of the thermotolerant *A. pasteurianus* 386B (13). They isolated this strain from cocoa bean heap fermentation with heating up to approximately 42–43°C (5). They also showed that the strain 386B rapidly produced acetic acid at a high concentration (17). We previously reported that SKU1108 is more thermotolerant than NBRC 3283 and performs acetic acid fermentation even at 38.5°C (22). Therefore, SKU1108 and 386B were defined as thermotolerant acetic acid fermentation strains. On the other hand, NBRC 3283 was defined as a mesophilic strain, which has the ability to perform fermentation at temperatures up to 37°C, but not at 38.5°C. Thus, in order to elucidate the mechanism underlying thermotolerance, a comparative genome analysis was performed using the complete genome sequences of one mesophilic and two thermotolerant strains. Whole genome level synteny was compared in these three complete genomes using Mauve aligner (Fig. S1). A genome synteny analysis revealed that the synteny of these three genomes was highly conserved.

**Fig. 1:**
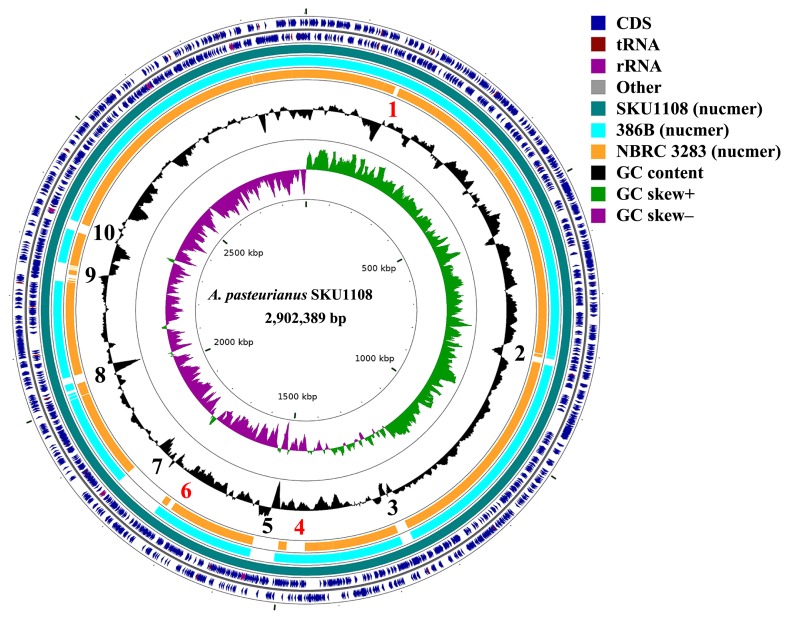
Comparison of complete genome sequences of three *Acetobacter pasteurianus* strains. Closely related genome sequences were aligned and mapped onto the complete genome sequence (AP014881) of the *A. pasteurianus* SKU1108 using NUCmer. The illustration was constructed using CG-view. Red and black colored numbers mean thermotolerant strains and SKU1108-specific regions, respectively.

In order to define the uniquely conserved genomic regions in the SKU1108 genome sequence, three complete genome sequences were mapped and aligned against the SKU1108 chromosome sequence using NUCmer, which is a part of the MUMmer genome alignment package. A circular map of the SKU1108 chromosome sequence with alignments is shown in Fig. 1. Ten strain-specific regions were identified (Table S1). Genes located in these regions are also shown in Table S2. Of these, regions 1, 4, and 6 were only conserved between the thermotolerant strains SKU1108 and 386B. Therefore, genes from these regions may be responsible for the thermotolerance phenotype of these strains and may be classified as thermotolerance-conferring conserved regions. Of the ten specific regions, regions 1, 2, 3, and 9 encode prophage-related proteins such as phage integrase and phage terminase. Therefore, these regions may have been acquired by prophage-insertion.

We also compared the repertoire of plasmid sequences among the three studied strains. SKU1108 had one large 187-kb plasmid 1 and three small plasmids (Table 1). On the other hand, 386B and NBRC 3283 had one (194 kb) and two (191 and 182 kb) large plasmids, respectively (2, 13). In order to clarify differences between the large plasmids, we aligned their sequences against the SKU1108 plasmid 1 sequence using NUCmer. Large plasmid 1 of SKU1108 and that of 386B, Apa386Bp1 (HF677571) were highly conserved (Fig. S2). Whole plasmid sequence comparisons also revealed that the synteny of these two plasmids was highly conserved (data not shown). In contrast, the two large plasmids of the strain NBRC 3283 were distinct from that of SKU1108 and not conserved. The results of the whole genome-based phylogenetic analysis suggested that SKU1108 and 386B are more closely related to each other than to NBRC 3283 (Fig. S3). Therefore, since the large plasmids were conserved in the two thermotolerant strains, they may have existed in the ancestor strain, *i.e.* before the divergence of SKU1108 and 386B into two separate thermotolerant strains.

### Genes associated with thermotolerance

In a previous study, we performed the thermal adaptation of SKU1108 by its repeated cultivation under acetic acid fermentation conditions. Consequently, two individually adapted strains, TI and TH-3, were obtained, and their mutational sites were identified using the draft SKU1108 genome assembly as a reference (22). In order to identify additional mutational sites, the previously reported Illumina sequence reads of two adapted strains were mapped onto the complete genomic DNA sequence of SKU1108 that also included the DNAs of four plasmids. No additional mutational sites were detected, indicating that the mapping analysis against the draft genome assembly constructed from Illumina reads was sufficient for a mutation site analysis of the adapted strains. All the mutational sites were detected on the chromosome sequence. Therefore, we have now changed the locus tag number based on the complete genome sequence (Table S3).

In order to identify the genes whose expression is required for growth at higher temperatures, we previously mutated the *A. tropicalis* SKU1100, a thermotolerant AAB that grows even at 42°C, by transposon mutagenesis, thereby inducing random insertions in the Tn*10* transposon using a conjugation method (30). We revealed that 24 “thermotolerant” genes (see Table 2 or Table S4) were associated with growth at higher temperatures. Therefore, in the present study, we clarified whether these 24 genes and their corresponding nucleotide and amino acid mutations were present in complete genomes. A phylogenetic analysis performed for each gene demonstrated that the thermotolerant genes from SKU1100 and three complete *A. pasteurianus* genomes were clustered in the same clade (data not shown). Therefore, together with the result that all thermotolerant genes were encoded in the conserved regions of all three complete genomes, we concluded that all known thermotolerant genes are conserved in the three genomes, even in the mesophilic strain NBRC 3283 (Table S4). The number of nucleotide/amino acid mutations in the 24 thermotolerant genes and additional 6 genes composed of operons with four genes (DNA methyltransferase [APT_00916], MinC [APT_00954], RpoE [APT_01242], and XdhA [APT_01267]) of the thermotolerant genes, *rhnB* (APT_00195), *minE* (APT_00952), *minD* (APT_00953), *rpoE* (APT_01243), *xdhC* (APT_01265), and *xdhB* (APT_01266) (30 genes in total) are shown in Table 2. The nucleotide sequences of the nine genes were identical in the three strains. There was some variation in nucleotide mutations in 21 other genes, 16 of which mainly accumulated nucleotide mutations in NBRC 3283 (Table 2 and Fig. S3). In contrast, the gene encoding asparagine synthetase (APT_01828) accumulated nucleotide mutations in SKU1108 only, and the gene for DNA methyltransferase in 386B only. In the remaining three genes, flavodoxin/nitric oxide synthase (APT_01927), *amiA* (APT_02041), and *cysG (*APT_02252), nucleotide mutations accumulated in all three strains. Non-synonymous mutations in these thermotolerant genes were also investigated and amino acid mutations were detected in 15 (Table 2). Of these, seven genes had amino acid mutations (10 mutations in total) that conferred substitutions by dissimilar amino acids. Amino acid mutations with dissimilar residues only occurred in NBRC 3283, suggesting that they are responsible for the less thermotolerant phenotype of NBRC 3283. Their mutation sites were APT_00246 (A115D), APT_00603 (S95R), APT_01265 (G106E), APT_01266 (T394R and E413G), APT_01267 (R161C, C162R, and T220I), APT_01828 (R471S), and APT_02252 (E185A). In particular, genes encoding the *xdhABC* operon (APT_01265–APT_01267) accumulated 6 amino acid mutations. Of these, the T220 residue (T206 in *Rhodobacter capsulatus*) of *xdhA* (APT_01267), shown to be in the active site in the crystal structure of *xdhAB* from *R. capsulatus*, is particularly important because it mediates hydrogen bonding with the co-factor FAD (36). Since mutated T220I cannot form hydrogen bonds with FAD, this mutation may affect the xanthine dehydrogenase activity of NBRC 3283.

In order to identify other thermotolerant genes only conserved in the two thermotolerant strains, we performed a BLASTP search against all proteins encoded in the three complete genomes using 24 genes of SKU1108 as the query. BLASTP hits (Table 3) showed that four thermotolerant genes, *lepA*, *ttg2C*, *xdhA*, and *degP*, had paralogs in the chromosome. However, a paralog of *xdhA* (APT_01390) encoding xanthine dehydrogenase XdhA (APT_01267) was only conserved in the two thermotolerant strains. This is a part of the *xdhAB* operon (APT_01390–APT_01389) that is encoded in specific region 4 found in the genomes of both thermotolerant strains (Fig. 1 and Table S2). Xanthine dehydrogenase is known to regulate heat shock and stress responses (30). This *xdhAB* paralog may partly contribute to the thermotolerance of SKU1108 and 386B. Thus, amino acid mutations accumulated in the thermotolerant genes and their paralogs encoded in the conserved thermotolerance-conferring region may be responsible for the thermotolerance of the two thermotolerant strains. As described above, two thermotolerant strains have the same large plasmid 1 (Fig. S2). Since these plasmids are only conserved in two thermotolerant strains, we speculated that the genes responsible for thermotolerance are encoded in these two large plasmids. Therefore, we also searched the paralog sequences encoded in these plasmid sequences by a BLASTP search. In addition, a BLASTP search was performed against all other plasmid sequences of the three strains. However, we were unable to identify any paralog sequences corresponding to the 24 thermotolerant genes in these plasmids.

Incidentally, related to the acetic acid resistance or acetic acid assimilation ability of *Acetobacter* species, we found an *aarC* paralog (*aarC1*) sequence in strain-specific region 7. *Acetobacter* species have a specialized citric acid cycle in which the *aarC* gene encoding acetate CoA-transferase plays a crucial role by regulating acetate assimilation (25). A phylogenetic analysis of the AarC protein, conserved in published *Acetobacter* genome sequences, produced the phylogenetic tree that was divided into two large clades: genes belonging to the *aarC* clade are conserved in all *Acetobacter* species, whereas those of the *aarC1* clade are only conserved in genomes of the three *A. pasteurianus* strains studied including SKU1108 (Fig. S4). Thus, it will be interesting to clarify the role of the *aarC1* gene in acetate assimilation.

## Conclusion

In the present study, we elucidated the complete genome sequence of the thermotolerant AAB, *A. pasteurianus* SKU1108, and compared it with those of other thermotolerant and mesophilic *A. pasteurianus* strains. By using a comparative genomic analysis of closely related strains, we revealed several candidate genes that underpin the thermotolerance phenotype. Further investigations of these closely related strains may provide novel insights into genetic causes of their specific phenotypes.

## Supplementary Information



## Figures and Tables

**Table 1:** Summary of the complete genome sequence of *Acetobacter pasteurianus* SKU1108

Genome information	Nucleotide sequence length (bp)	GC content (%)	Number of ORFs	Number of tRNAs	Number of tmRNAs	Number of rRNAs	GenBank accession number
chromosome	2,902,389	52.79	2,662	56	1	15	AP014881
plasmid 1	187,193	52.02	201	—	—	—	AP014882
plasmid 2	6,331	52.88	8	—	—	—	AP014883
plasmid 3	2,799	58.20	3	—	—	—	AP014884
plasmid 4	2,278	55.71	2	—	—	—	AP014885
Total	3,135,082	52.75	2,876	56	1	15 (5 sets of the rRNA operon)	—

**Table 2:** Number of nucleotide and amino acid mutations of 24 thermotolerant genes among three analyzed strains

Gene	Product	Locus_tag	Nucleotide mutation			Amino acid mutation		
								
SKU1108	SKU1108	SKU1108	NBRC 3283	386B	SKU1108	NBRC 3283	386B	SKU1108
—	hypothetical protein	APT_00053	0	0	0	0	0	0
*hpnB*	glycosyl transferase	APT_00180	0	0	0	0	0	0
*poxA*	lysyl-tRNA synthetase	APT_00246	13	0	0	2 (1)	0	0
*lepA*	GTP-binding protein LepA	APT_00584	9	0	0	1 (0)	0	0
—	hypothetical protein	APT_00590	4	0	0	0	0	0
*mmpA*	putative metalloprotease MmpA	APT_00603	8	0	0	1 (1)	0	0
*hsp20*	heat shock protein Hsp20	APT_00623	0	0	0	0	0	0
—	hypothetical protein	APT_00856	5	0	0	0	0	0
*glnE*	glutamate-ammonia-ligase adenylyltransferase	APT_00857	0	0	0	0	0	0
*rnhB*	ribonuclease HII	APT_00915	0	0	0	0	0	0
*yhdJ*	DNA methyltransferase	APT_00916	0	1	0	0	0	0
*minE*	cell division topological specificity factor MinE	APT_00952	3	0	0	2 (0)	0	0
*minD*	cell division inhibitor MinD	APT_00953	9	0	0	0	0	0
*minC*	cell division inhibitor MinC	APT_00954	4	0	0	1 (0)	0	0
—	hypothetical protein	APT_00961	16	0	0	0	0	0
*nhaK2*	Na^+^/H^+^ antiporter	APT_00984	69	0	0	1 (0)	0	0
*smc*	chromosome segregation protein SMC	APT_01022	44	0	0	9 (0)	0	0
*ttg2C*	toluene ABC transporter periplasmic protein	APT_01083	0	0	0	0	0	0
*serA*	d-3-phosphoglycerate dehydrogenase	APT_01136	6	0	0	0	0	0
*rpoE*	RNA polymerase sigma-E factor (sigma-24) protein 2	APT_01242	0	0	0	0	0	0
*rpoE*	RNA polymerase sigma-E factor (sigma-24) protein 1	APT_01243	0	0	0	0	0	0
*xdhC*	xanthine dehydrogenase accessory protein XdhC	APT_01265	7	1	0	1 (1)	1 (0)	0
*xdhB*	xanthine dehydrogenase molybdopterin binding subunit XdhB	APT_01266	48	0	0	9 (2)	0	0
*xdhA*	xanthine dehydrogenase XdhA	APT_01267	28	0	0	11 (3)	0	0
—	asparagine synthetase	APT_01828	1	1	30	1 (1)	1 (0)	5 (0)
—	flavodoxin/nitric oxide synthase	APT_01927	5	5	4	2 (0)	1 (0)	1 (0)
*amiA*	*N*-acetylmuramoyl-l-alanine amidase	APT_02041	11	8	9	2 (0)	0	1 (0)
*cysG*	siroheme synthase	APT_02252	16	5	8	3 (1)	0	2 (0)
—	hypothetical protein	APT_02411	0	0	0	0	0	0
*htrA*	endopeptidase DegP/Do	APT_02556	21	0	0	3 (0)	0	0

Numbers in parentheses indicate residues substituted with dissimilar amino acids. An additional six genes: APT_00915, APT_00952, APT_00953, APT_01243, APT_01265, and APT_01266, which are clustered with 24 thermotolerant genes, are also included in the mutational analysis.

**Table 3:** List of paralogous sequences of 24 thermotolerant genes conserved in three *A. pasteurianus* strains

Gene SKU1108	Blast query	Blast hits	Gene (hits)	Product (hits)	E-value	Identity (%)
*lepA*	APT_00584	APT_00570	*typA*	GTP-binding protein TypA	5.00E-32	26
		APA01_06200	*bipA*	GTP-binding protein TypA	5.00E-32	26
		APA386B_2113	*typA*	GTP-binding protein TypA/BipA	5.00E-32	26

*ttg2C*	APT_01083	APA386B_1720	*ttg2C*	toluene transporter substrate-binding periplasmic protein Ttg2C	4.00E-14	35
		APT_00211	*ttg2C*	toluene ABC transporter periplasmic protein	4.00E-14	35
		APA01_02300	*ttg2C*	toluene ABC transporter periplasmic protein	4.00E-14	35

*xdhA*	APT_01267	APA386B_251	*xdhA*	xanthine dehydrogenase small subunit	4.00E-53	32
		APT_01390	*xdhA*	xanthine dehydrogenase XdhA	4.00E-53	32

*htrA*	APT_02556	APA386B_1296	*degP*	endopeptidase DegP/Do	1.00E-94	42
		APT_02481	*degP*	endopeptidase DegP/Do	1.00E-94	42
		APA01_24860	*degP*	endopeptidase DegP/Do	2.00E-94	42
