# Supplementary material for: Complete Genome Sequencing and Comparative Genomic Analysis of the Thermotolerant Acetic Acid Bacterium, Acetobacter pasteurianus SKU1108, Provide a New Insight into Thermotolerance
Source: Microbes Environ. 2016 Sep 24;31(4):395–400. doi: 10.1264/jsme2.ME16023 (PMC5158111; doi:10.1264/jsme2.ME16023)
Supplement: Supplementary file 1 [file 31_395_s1.pdf]

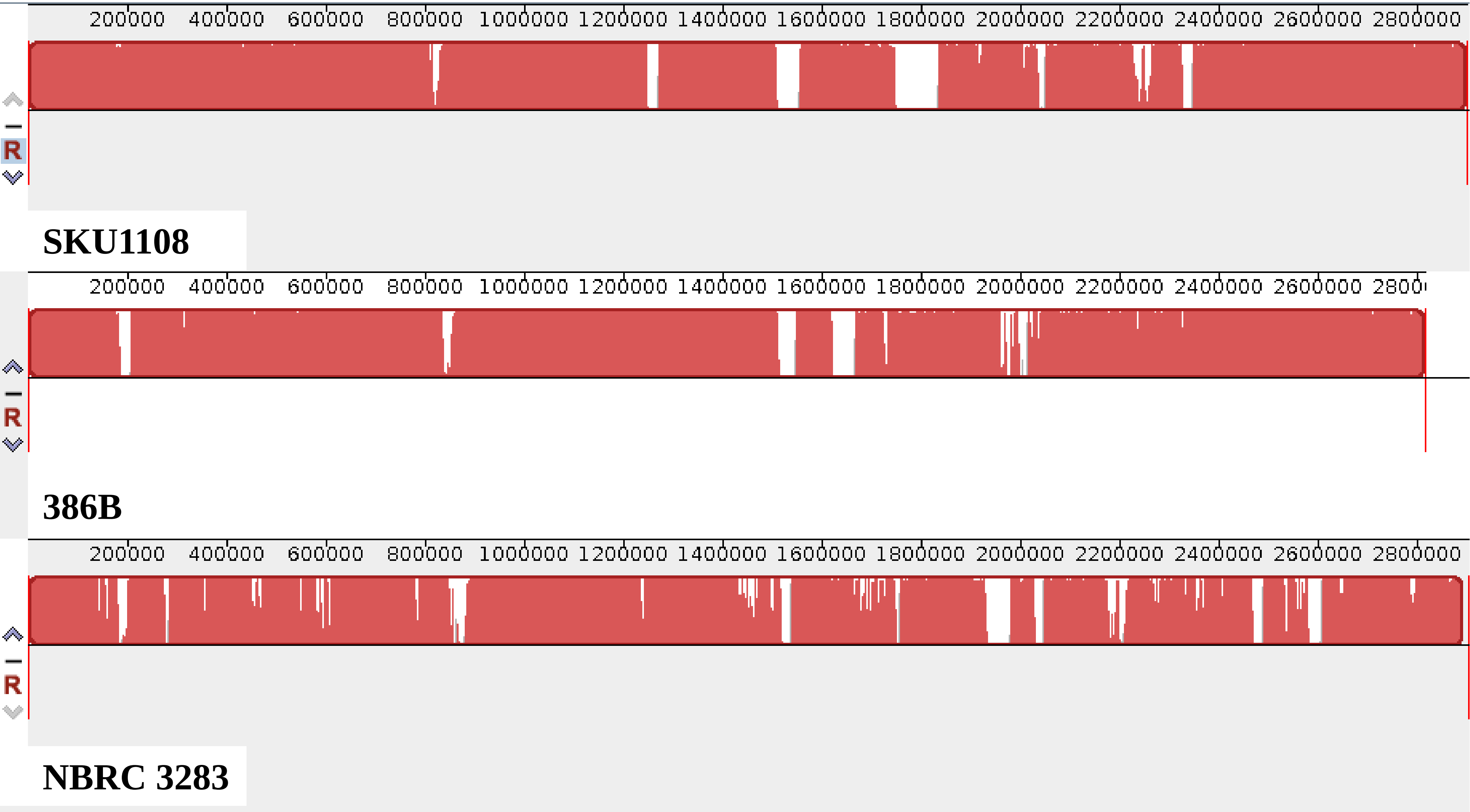

Fig. S1. Alignment of the *A. pasteurianus* SKU1108 genome to *A. pasteurianus* 386B, and *A. pasteurianus* NBRC 3283. Alignments were generated using progressiveMauve program with default parameter (Altschul *et al*. 1997. Nucleic Acids Res. 25:3389–3402).

Fig. S1 Matsutani et al., 2016

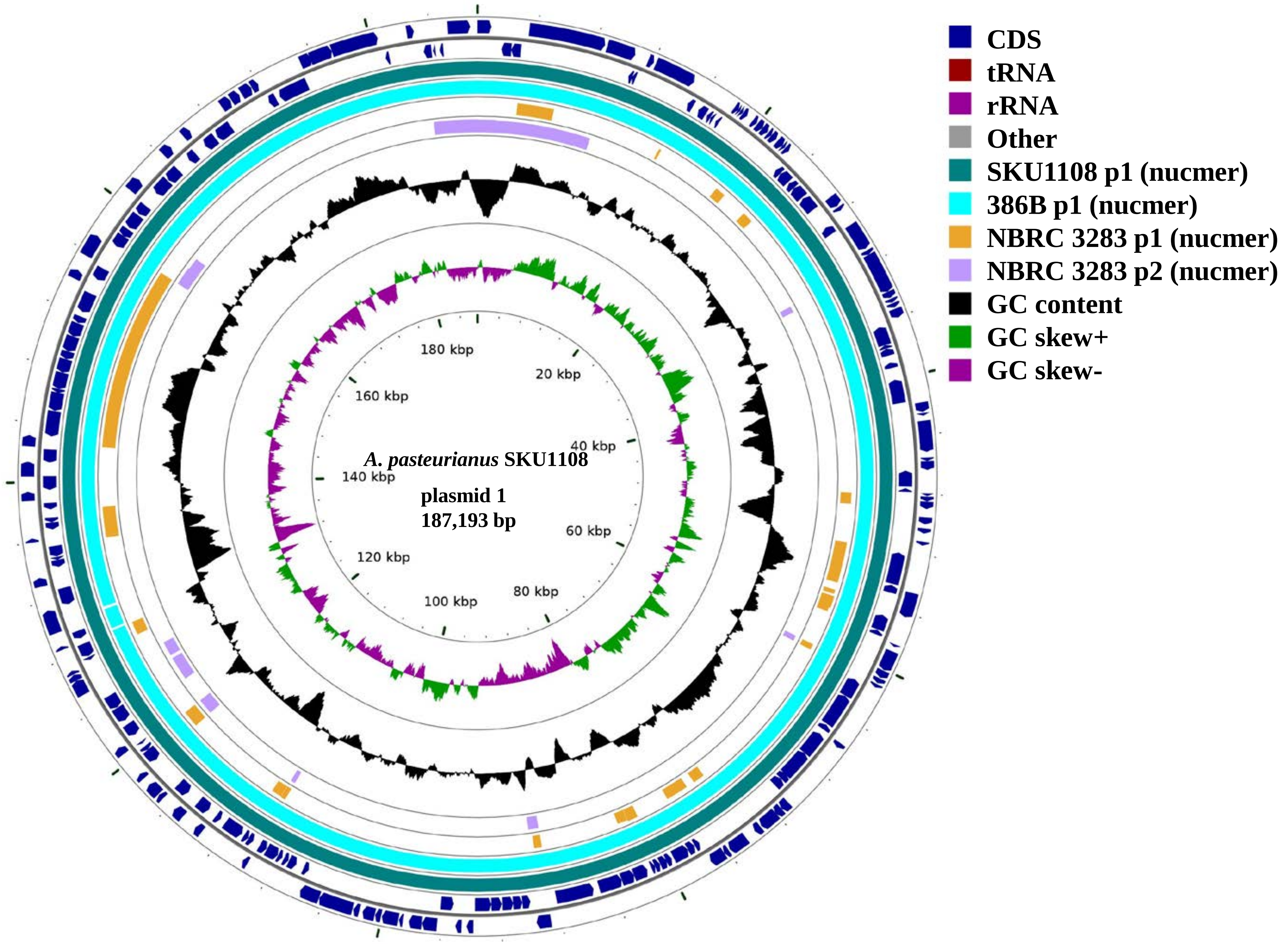

Fig. S2 Comparison view of the four large plasmid sequences of three strains. Plasmid sequences, SKU1108 (AP014882), 386B (HF677571), and NBRC 3283 (AP011122 and AP011123) were aligned and mapped onto large plasmid of SKU1108 (AP014882) using NUCmer. Figure was constructed by using CG-view.

Fig. S2 Matsutani et al., 2016

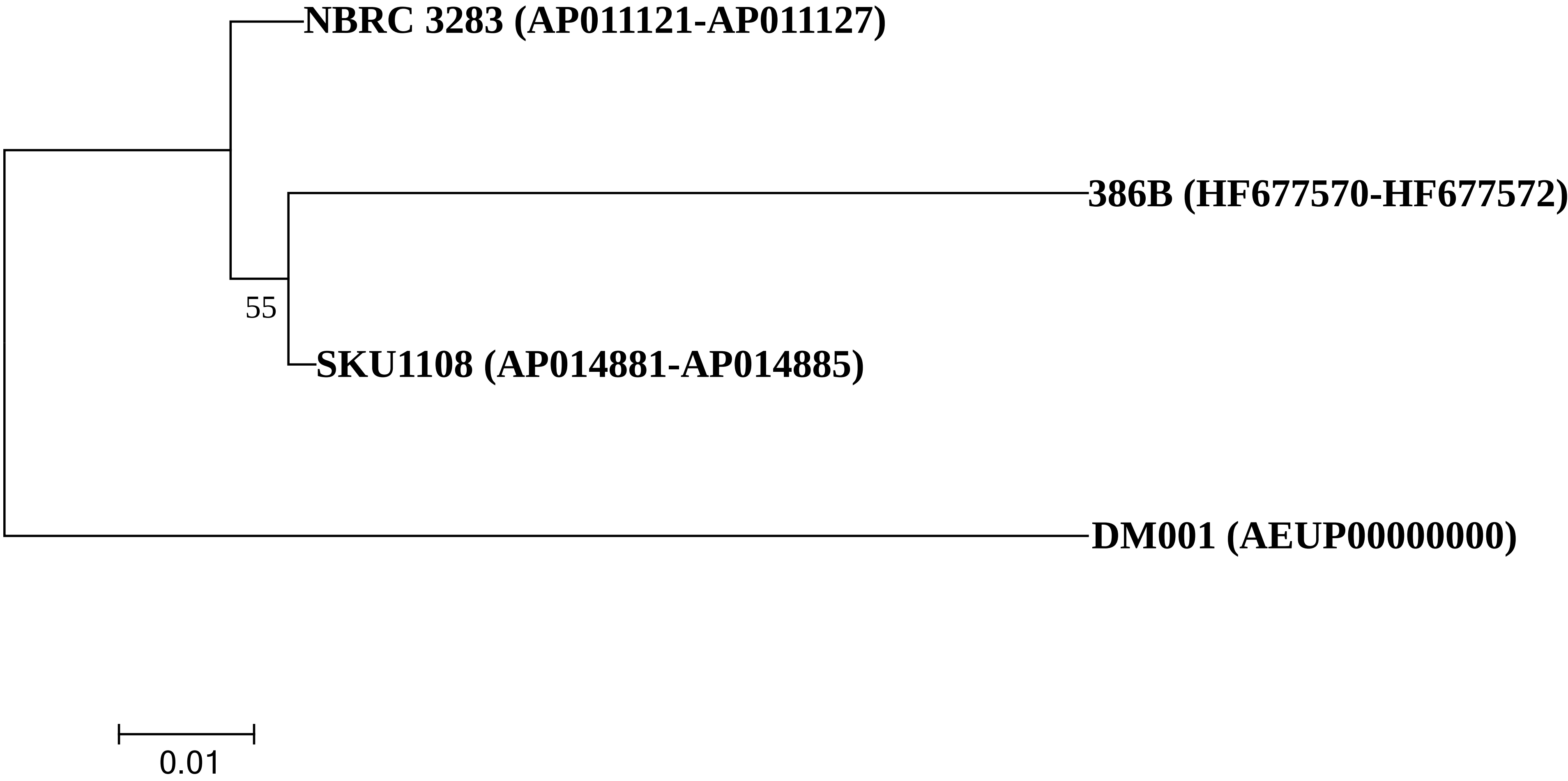

Fig. S3 Maximum-likelihood genome-based phylogenetic tree calculated with MUSCLE from 1,895 orthologous nucleotide sequences of *Acetobacter pasteurianus*. The orthologous set was identified as previously reported (Matsutani *et al*. 2015. Appl. Microbiol. Biotechnol. 99:7229–7240). The gene-support frequency (GSF) is shown (Suzuki *et al*. 2012. BMC Genomics 13:38). Draft genome sequence of *Acetobacter pomorum* DM001 strain was used as outgroup.

Fig. S3 Matsutani et al., 2016

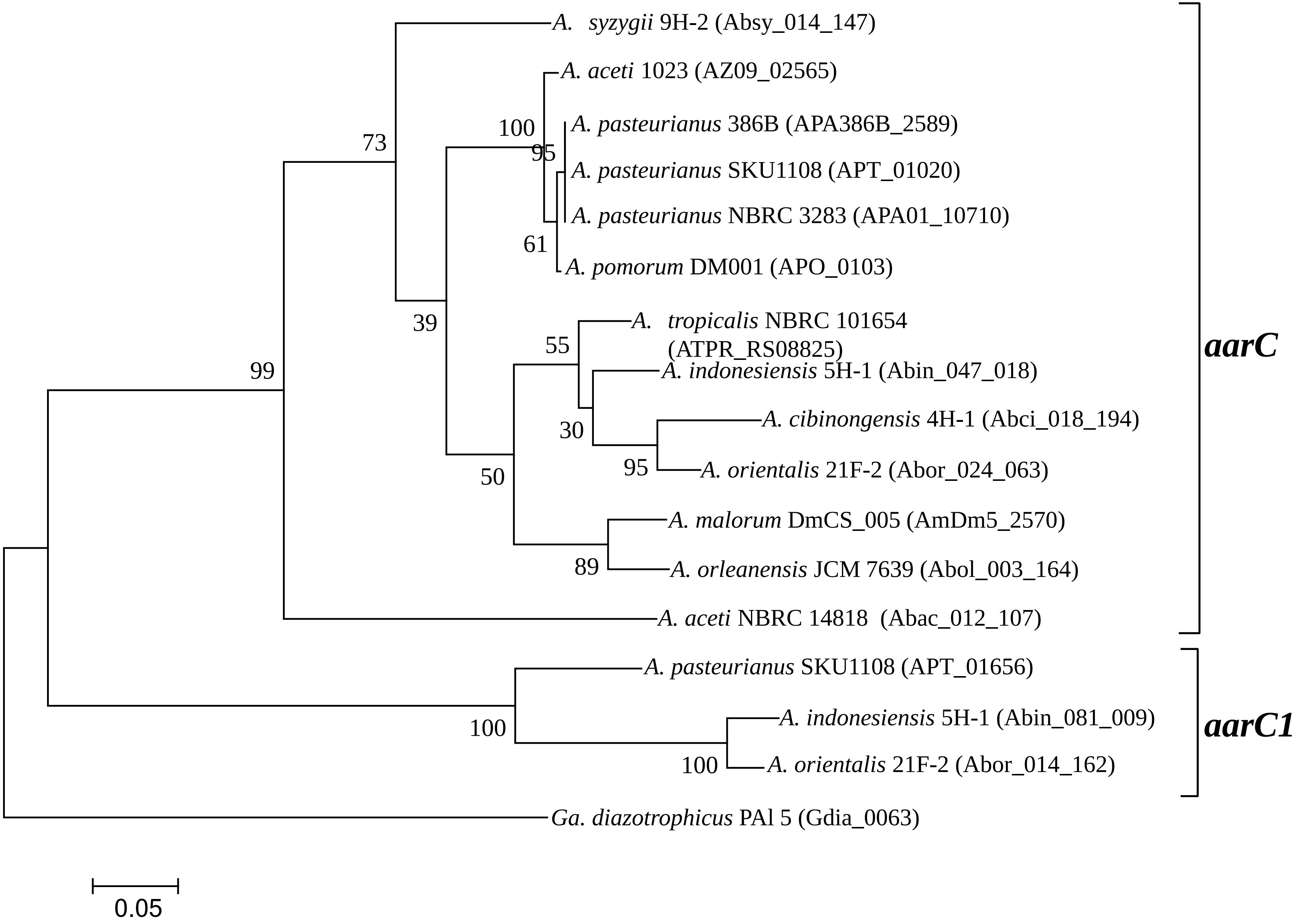

Fig. S4 Maximum-likelihood phylogenetic trees of selected *Acetobacter* species strains and their AarC proteins. The tree was prepared based on amino acid sequences of *aarC* gene of the 14 genomes. *Gluconacetobacter diazotrophicus* Pal 5 was used as an outgroup. Bootstrap values with 1,000 bootstrap replicates are shown. Locus tag of *aarC* genes are also shown.

Fig. S4 Matsutani et al., 2016

Table S1. List of specific region of SKU1108. Two genomes, 386B and NBRC 3283, were mapped onto SKU1108 chromosome by using NUCmer. Unmapped regions of SKU1108 using each genome are shown. Specific region 1,4 and 6 is also conserved in 386B genome.

	386B		NBRC 3283	
specific region	initial	end	initial	end
specific region 1	-	-	158468	184478
specific region 2	816475	828978	817153	824283
specific region 3	1248995	1269982	1248995	1269982
specific region 4	-	-	1453808	1489478
specific region 5	1510344	1555927	1505616	1555929
specific region 6	-	-	1726953	1737279
specific region 7	1748925	1835024	1749641	1835024
specific region 8	2037496	2049733	2032875	2049733
specific region 9	2231021	2265703	2238986	2246200
specific region 9-1	-	-	2255003	2256312
specific region 10	2328919	2346998	2328799	2346998

Table S2. List of genes encoded in specific region of SKU1108. Specific region 1,4 and 6 is also conserved in 386B genome.

specific region	locus_tag	gene	product	GenBank accession No.	start	end
specific region 1	APT_00139	-	hypothetical protein	AP014881	160750	157595
specific region 1	APT_00140	-	glycosyl transferase	AP014881	162027	160756
specific region 1	APT_00141	-	hypothetical protein	AP014881	162981	162472
specific region 1	APT_00142	-	hypothetical protein	AP014881	164210	162981
specific region 1	APT_00143	*amiF*	formamidase	AP014881	164589	165596
specific region 1	APT_00144	*luxR*	LuxR family transcriptional regulator	AP014881	166542	165643
specific region 1	APT_00145	-	two-component hybrid sensor and regulator	AP014881	170021	166539
specific region 1	APT_00146	*urtA*	urea ABC transporter periplasmic protein	AP014881	170022	171359
specific region 1	APT_00147	*urtB*	urea ABC transporter permease	AP014881	171414	172340
specific region 1	APT_00148	*urtC*	urea ABC transporter permease	AP014881	172352	173467
specific region 1	APT_00149	*urtD*	urea ABC transporter ATP-binding protein	AP014881	173479	174225
specific region 1	APT_00150	*urtE*	urea ABC transporter ATP-binding protein	AP014881	174242	174958
specific region 1	APT_00151	-	hypothetical protein	AP014881	175590	175159
specific region 1	APT_00152	-	putative phage integrase	AP014881	176026	175772
specific region 1	APT_00153	-	hypothetical protein	AP014881	175796	176230
specific region 1	APT_00154	-	hypothetical protein	AP014881	178202	176394
specific region 1	APT_00155	-	hypothetical protein	AP014881	178580	178227
specific region 1	APT_00156	*cpo*	non-heme chloroperoxidase	AP014881	179754	178918
specific region 1	APT_00157	-	transcriptional regulator MerR	AP014881	179836	180363
specific region 1	APT_00158	-	protease/amidase	AP014881	181052	180360
specific region 1	APT_00159	-	hypothetical protein	AP014881	181203	182123
specific region 1	APT_00160	-	hypothetical protein	AP014881	182413	183375
specific region 1	APT_00161	-	hypothetical protein	AP014881	183350	183733
specific region 1	APT_00162	-	phage integrase	AP014881	185447	183624
specific region 2	APT_00776	-	hypothetical protein	AP014881	818517	817801
specific region 2	APT_00777	-	hypothetical protein	AP014881	818586	818993
specific region 2	APT_00778	-	hypothetical protein	AP014881	818938	820053
specific region 2	APT_00779	-	hypothetical protein	AP014881	820057	820155
specific region 2	APT_00780	-	hypothetical protein	AP014881	821007	820432
specific region 2	APT_00781	-	uncharacterized phage-associated protein-like protein	AP014881	821509	821000
specific region 2	APT_00782	-	prophage integrase	AP014881	823304	822042
specific region 3	APT_01192	-	phage integrase	AP014881	1249079	1250281
specific region 3	APT_01193	-	hypothetical protein	AP014881	1250396	1251232
specific region 3	APT_01194	-	phage associated-antirepressor BRO	AP014881	1252135	1251380
specific region 3	APT_01195	-	hypothetical protein	AP014881	1252503	1252132
specific region 3	APT_01196	-	hypothetical protein	AP014881	1252855	1253754
specific region 3	APT_01197	-	hypothetical protein	AP014881	1255147	1253840
specific region 3	APT_01198	-	hypothetical protein	AP014881	1255459	1255250
specific region 3	APT_01199	-	hypothetical protein	AP014881	1256228	1255536
specific region 3	APT_01200	-	hypothetical protein	AP014881	1256458	1256294
specific region 3	APT_01201	-	phage related lysozyme	AP014881	1256953	1256495
specific region 3	APT_01202	-	hypothetical protein	AP014881	1257342	1257130
specific region 3	APT_01203	-	terminase	AP014881	1258088	1257375
specific region 3	APT_01204	-	prophage terminase small subunit	AP014881	1258674	1258078
specific region 3	APT_01205	-	endonuclease	AP014881	1259203	1258874
specific region 3	APT_01206	-	hypothetical protein	AP014881	1259832	1259200
specific region 3	APT_01207	-	phage DNA recombinase	AP014881	1260745	1259870
specific region 3	APT_01208	-	hypothetical protein	AP014881	1261306	1260896
specific region 3	APT_01209	-	hypothetical protein	AP014881	1262171	1261443
specific region 3	APT_01210	-	hypothetical protein	AP014881	1262703	1262200
specific region 3	APT_01211	-	hypothetical protein	AP014881	1262975	1262724
specific region 3	APT_01212	-	hypothetical protein	AP014881	1264129	1262972
specific region 3	APT_01213	-	hypothetical protein	AP014881	1264616	1264122
specific region 3	APT_01214	-	hypothetical protein	AP014881	1265090	1264632
specific region 3	APT_01215	-	hypothetical protein	AP014881	1265368	1265763
specific region 3	APT_01216	-	hypothetical protein	AP014881	1265955	1266512
specific region 3	APT_01217	-	hypothetical protein	AP014881	1266766	1267023
specific region 3	APT_01218	-	hypothetical protein	AP014881	1267020	1267250
specific region 3	APT_01219	-	hypothetical protein	AP014881	1267250	1267471
specific region 3	APT_01220	-	RelA/SpoT protein	AP014881	1268637	1267642
specific region 3	APT_01221	-	putative phage associated protein	AP014881	1269707	1268634
specific region 4	APT_01365	*pigA*	heme oxygenase	AP014881	1453538	1454104
specific region 4	APT_01366	-	hypothetical protein	AP014881	1454522	1454178
specific region 4	APT_01367	*hmuV*	hemin importer ATP-binding subunit	AP014881	1455331	1454522
specific region 4	APT_01368	*hmuU*	transmembrane permease component of haem ABC transporter	AP014881	1456383	1455328
specific region 4	APT_01369	*hmuT*	hemin ABC transporter hemin-binding protein	AP014881	1457366	1456380
specific region 4	APT_01370	-	hypothetical protein	AP014881	1457734	1457366
specific region 4	APT_01371	*mntR*	manganese transport regulator MntR	AP014881	1458401	1457919
specific region 4	APT_01372	*mntH*	manganese transport protein MntH	AP014881	1458587	1459960
specific region 4	APT_01373	-	hypothetical protein	AP014881	1460103	1460384
specific region 4	APT_01374	-	hypothetical protein	AP014881	1461041	1460442
specific region 4	APT_01375	-	hypothetical protein	AP014881	1461347	1461901
specific region 4	APT_01376	-	hypothetical protein	AP014881	1462443	1463585
specific region 4	APT_01377	-	alpha/beta hydrolase	AP014881	1463582	1464610
specific region 4	APT_01378	*amt*	ammonium transporter	AP014881	1465964	1464663
specific region 4	APT_01379	-	hypothetical protein	AP014881	1466264	1466010
specific region 4	APT_01380	*tynA*	tyramine oxidase	AP014881	1468535	1466331

specific region 4	APT_01381	-	hypothetical protein	AP014881	1468502	1469512
specific region 4	APT_01382	-	hypothetical protein	AP014881	1469853	1469521
specific region 4	APT_01383	-	low-affinity zinc transport protein	AP014881	1471078	1469846
specific region 4	APT_01384	-	NADH:flavin oxidoreductase	AP014881	1472859	1471786
specific region 4	APT_01385	-	short-chain dehydrogenase/reductase SDR	AP014881	1473700	1472954
specific region 4	APT_01386	-	AraC family transcriptional regulator	AP014881	1473854	1474768
specific region 4	APT_01387	-	metallophosphoesterase	AP014881	1475953	1474838
specific region 4	APT_01388	-	TetR family transcriptional regulator	AP014881	1476772	1476152
specific region 4	APT_01389	*xdhB*	xanthine dehydrogenase XdhB	AP014881	1476885	1479017
specific region 4	APT_01390	*xdhA*	xanthine dehydrogenase XdhA	AP014881	1479014	1480342
specific region 4	APT_01391	-	outer membrane protein	AP014881	1482388	1480421
specific region 4	APT_01392	-	outer membrane protein	AP014881	1484281	1482527
specific region 4	APT_01393	-	bacteriocin/lantibiotic exporter permease	AP014881	1486927	1484750
specific region 4	APT_01394	*eamA*	transporter EamA	AP014881	1488291	1486927
specific region 4	APT_01395	-	hypothetical protein	AP014881	1488768	1488367
specific region 4	APT_01396	-	hypothetical protein	AP014881	1488896	1488783
specific region 5	APT_01415	*hsdS*	type I restriction-modification enzyme S subunit	AP014881	1510325	1511524
specific region 5	APT_01416	*hsdR*	type I site-specific deoxyribonuclease	AP014881	1511554	1514850
specific region 5	APT_01417	-	metal-dependent hydrolase	AP014881	1514853	1515590
specific region 5	APT_01418	-	hypothetical protein	AP014881	1515787	1515599
specific region 5	APT_01419	-	glycosyl transferase	AP014881	1517242	1516082
specific region 5	APT_01420	-	beta-ketoacyl synthase	AP014881	1519032	1517242
specific region 5	APT_01421	-	polyketide synthase	AP014881	1525325	1519035
specific region 5	APT_01422	-	probable long-chain fatty-acid-CoA ligase	AP014881	1526829	1525354
specific region 5	APT_01423	-	outer membrane protein	AP014881	1529698	1526918
specific region 5	APT_01424	-	hypothetical protein	AP014881	1529946	1530227
specific region 5	APT_01425	-	transposase	AP014881	1530288	1530635
specific region 5	APT_01426	-	IS4 family transposase	AP014881	1530656	1531045
specific region 5	APT_01427	-	hypothetical protein	AP014881	1536520	1531055
specific region 5	APT_01428	-	hypothetical protein	AP014881	1538160	1536517
specific region 5	APT_01429	-	protein kinase	AP014881	1542302	1538166
specific region 5	APT_01430	-	type I restriction-modification system S subunit	AP014881	1543711	1542302
specific region 5	APT_01431	-	type I restriction-modification system, M subunit, putative	AP014881	1545732	1543708
specific region 5	APT_01432	*kdpB*	potassium-transporting ATPase subunit B	AP014881	1547777	1545729
specific region 5	APT_01433	-	transcriptional regulator	AP014881	1548103	1548459
specific region 5	APT_01434	-	IS4 family transposase	AP014881	1548883	1548494
specific region 5	APT_01435	-	transposase	AP014881	1549251	1548904
specific region 5	APT_01436	-	hypothetical protein	AP014881	1549482	1550297
specific region 5	APT_01437	-	hypothetical protein	AP014881	1551832	1550426
specific region 5	APT_01438	-	lytic transglycosylase	AP014881	1552697	1552026
specific region 5	APT_01439	-	hypothetical protein	AP014881	1553035	1552694
specific region 5	APT_01440	-	conjugal transfer protein TraF	AP014881	1553318	1553094
specific region 5	APT_01441	-	IS4 family transposase	AP014881	1553735	1553346
specific region 5	APT_01442	-	transposase	AP014881	1554103	1553756
specific region 5	APT_01443	-	putative DNA recombinase	AP014881	1554290	1555756
specific region 6	APT_01586	-	outer membrane protein	AP014881	1727849	1725477
specific region 6	APT_01587	-	hypothetical protein	AP014881	1728314	1727922
specific region 6	APT_01588	*lysR*	LysR family transcriptional regulator	AP014881	1728687	1729634
specific region 6	APT_01589	-	TonB-dependent siderophore receptor	AP014881	1731990	1729684
specific region 6	APT_01590	-	hypothetical protein	AP014881	1732675	1732325
specific region 6	APT_01591	-	hypothetical protein	AP014881	1734225	1732672
specific region 6	APT_01592	-	hypothetical protein	AP014881	1734513	1734229
specific region 6	APT_01593	*ufrA*	TonB-dependent siderophore receptor	AP014881	1737167	1734513
specific region 7	APT_01606	-	transposase	AP014881	1749701	1750048
specific region 7	APT_01607	-	IS4 family transposase	AP014881	1750069	1750458
specific region 7	APT_01608	-	hypothetical protein	AP014881	1751785	1750616
specific region 7	APT_01609	-	hypothetical protein	AP014881	1752066	1752173
specific region 7	APT_01610	-	transcriptional regulator	AP014881	1752250	1752792
specific region 7	APT_01611	-	Cupin 2 conserved barrel domain protein	AP014881	1752828	1753217
specific region 7	APT_01612	-	hypothetical protein	AP014881	1753239	1754147
specific region 7	APT_01613	-	cupin 2 domain-containing protein	AP014881	1754704	1754333
specific region 7	APT_01614	-	major facilitator superfamily AmpG-like permease	AP014881	1755906	1754701
specific region 7	APT_01615	-	hypothetical protein	AP014881	1758036	1756003
specific region 7	APT_01616	-	regulator protein PchR	AP014881	1758223	1759107
specific region 7	APT_01617	*xylP*	xylose-proton symporter	AP014881	1760711	1759407
specific region 7	APT_01618	-	hypothetical protein	AP014881	1761689	1761132
specific region 7	APT_01619	-	hypothetical protein	AP014881	1762246	1761923
specific region 7	APT_01620	-	hypothetical protein	AP014881	1763513	1762599
specific region 7	APT_01621	-	hypothetical protein	AP014881	1763525	1763812
specific region 7	APT_01622	*ntrR*	transcriptional regulator NtrR	AP014881	1764311	1763901
specific region 7	APT_01623	-	antitoxin	AP014881	1764536	1764312
specific region 7	APT_01624	-	hypothetical protein	AP014881	1764714	1764980
specific region 7	APT_01625	-	hypothetical protein	AP014881	1765599	1766636
specific region 7	APT_01626	-	transposase	AP014881	1766729	1767019
specific region 7	APT_01627	-	DEAD/DEAH box helicase-like	AP014881	1770205	1767626
specific region 7	APT_01628	-	hypothetical protein	AP014881	1770795	1770202
specific region 7	APT_01629	-	restriction endonuclease	AP014881	1771250	1771945
specific region 7	APT_01630	-	acetyltransferase	AP014881	1772437	1773078
specific region 7	APT_01631	-	hypothetical protein	AP014881	1773603	1773953
specific region 7	APT_01632	-	hypothetical protein	AP014881	1773944	1774693
specific region 7	APT_01633	-	hypothetical protein	AP014881	1774839	1775594
specific region 7	APT_01634	-	hypothetical protein	AP014881	1775636	1775887

specific region 7	APT_01635	-	hypothetical protein	AP014881	1775988	1776230
specific region 7	APT_01636	-	hypothetical protein	AP014881	1776836	1777993
specific region 7	APT_01637	-	endoribonuclease L-PSP	AP014881	1779135	1778647
specific region 7	APT_01638	-	MerR family transcriptional regulator	AP014881	1779154	1779672
specific region 7	APT_01639	-	hypothetical protein	AP014881	1779748	1779993
specific region 7	APT_01640	-	hypothetical protein	AP014881	1780243	1781448
specific region 7	APT_01641	-	transposase	AP014881	1781562	1781885
specific region 7	APT_01642	-	transposase	AP014881	1781882	1782772
specific region 7	APT_01643	-	hypothetical protein	AP014881	1782849	1784465
specific region 7	APT_01644	-	hypothetical protein	AP014881	1784724	1784443
specific region 7	APT_01645	-	hypothetical protein	AP014881	1785141	1785986
specific region 7	APT_01646	-	2-methylthioadenine synthetase	AP014881	1785986	1786219
specific region 7	APT_01647	-	transposase	AP014881	1787106	1786216
specific region 7	APT_01648	-	transposase	AP014881	1787426	1787103
specific region 7	APT_01649	-	hypothetical protein	AP014881	1787504	1787782
specific region 7	APT_01650	-	hypothetical protein	AP014881	1788252	1788593
specific region 7	APT_01651	-	hypothetical protein	AP014881	1788854	1789942
specific region 7	APT_01652	-	hypothetical protein	AP014881	1790705	1790166
specific region 7	APT_01653	-	hypothetical protein	AP014881	1792331	1791369
specific region 7	APT_01654	-	AraC family transcriptional regulator	AP014881	1793122	1792328
specific region 7	APT_01655	-	AraC family transcriptional regulator	AP014881	1794631	1793666
specific region 7	APT_01656	*aarC1*	acetyl-CoA hydrolase	AP014881	1796162	1794651
specific region 7	APT_01657	-	hypothetical protein	AP014881	1796535	1796669
specific region 7	APT_01658	-	hypothetical protein	AP014881	1796669	1797226
specific region 7	APT_01659	-	multidrug resistance transporter EmrB/QacA	AP014881	1798028	1797423
specific region 7	APT_01660	-	hypothetical protein	AP014881	1798187	1799236
specific region 7	APT_01661	-	hypothetical protein	AP014881	1799196	1800737
specific region 7	APT_01662	-	hypothetical protein	AP014881	1801132	1800872
specific region 7	APT_01663	-	hypothetical protein	AP014881	1802300	1802136
specific region 7	APT_01664	-	hypothetical protein	AP014881	1802411	1803343
specific region 7	APT_01665	-	transposase	AP014881	1803340	1803663
specific region 7	APT_01666	-	transposase	AP014881	1803660	1804550
specific region 7	APT_01667	-	hypothetical protein	AP014881	1804619	1804852
specific region 7	APT_01668	-	transposase	AP014881	1805622	1805194
specific region 7	APT_01669	-	hypothetical protein	AP014881	1806824	1805619
specific region 7	APT_01670	-	transposase	AP014881	1807848	1806979
specific region 7	APT_01671	-	transposase	AP014881	1808165	1807896
specific region 7	APT_01672	-	transposase	AP014881	1808982	1808245
specific region 7	APT_01673	-	hypothetical protein	AP014881	1810300	1809308
specific region 7	APT_01674	-	hypothetical protein	AP014881	1811211	1810297
specific region 7	APT_01675	-	hypothetical protein	AP014881	1812071	1811268
specific region 7	APT_01676	-	methyltransferase type 11	AP014881	1812948	1812202
specific region 7	APT_01677	-	hypothetical protein	AP014881	1813568	1813062
specific region 7	APT_01678	-	hypothetical protein	AP014881	1813927	1813640
specific region 7	APT_01679	-	hypothetical protein	AP014881	1814066	1813920
specific region 7	APT_01680	-	hypothetical protein	AP014881	1814326	1814090
specific region 7	APT_01681	-	phage repressor	AP014881	1814479	1815159
specific region 7	APT_01682	-	hypothetical protein	AP014881	1815622	1815957
specific region 7	APT_01683	-	DNA topoisomerase I	AP014881	1817397	1816435
specific region 7	APT_01684	-	exodeoxyribonuclease VII large subunit	AP014881	1817814	1819739
specific region 7	APT_01685	-	hypothetical protein	AP014881	1819742	1819978
specific region 7	APT_01686	-	hypothetical protein	AP014881	1820354	1820115
specific region 7	APT_01687	-	hypothetical protein	AP014881	1820694	1820329
specific region 7	APT_01688	-	ankyrin	AP014881	1821191	1820973
specific region 7	APT_01689	*araC*	transcriptional regulator AraC/YbtA	AP014881	1821609	1822571
specific region 7	APT_01690	-	sulfotransferase	AP014881	1823391	1822561
specific region 7	APT_01691	-	hypothetical protein	AP014881	1824318	1823578
specific region 7	APT_01692	-	acyltransferase	AP014881	1824528	1826501
specific region 7	APT_01693	-	hypothetical protein	AP014881	1827195	1826638
specific region 7	APT_01694	-	hypothetical protein	AP014881	1827902	1827198
specific region 7	APT_01695	-	hypothetical protein	AP014881	1828196	1827990
specific region 7	APT_01696	-	hypothetical protein	AP014881	1829154	1828501
specific region 7	APT_01697	-	hypothetical protein	AP014881	1829798	1829160
specific region 7	APT_01698	-	hypothetical protein	AP014881	1831136	1831480
specific region 7	APT_01699	-	hypothetical protein	AP014881	1831710	1832888
specific region 7	APT_01700	-	hypothetical protein	AP014881	1832959	1833150
specific region 8	APT_01882	-	hypothetical protein	AP014881	2037725	2037865
specific region 8	APT_01883	-	transposase	AP014881	2038555	2037860
specific region 8	APT_01884	-	hypothetical protein	AP014881	2040360	2039008
specific region 8	APT_01885	-	transposase	AP014881	2041116	2040847
specific region 8	APT_01886	-	transposase	AP014881	2041682	2041209
specific region 8	APT_01887	-	hypothetical protein	AP014881	2042902	2041916
specific region 8	APT_01888	-	hypothetical protein	AP014881	2044560	2043190
specific region 8	APT_01889	-	hypothetical protein	AP014881	2046993	2044882
specific region 8	APT_01890	-	outer membrane protein	AP014881	2048946	2047225
specific region 9	APT_02058	-	phage-related minor tail protein	AP014881	2242225	2236976
specific region 9	APT_02059	-	hypothetical protein	AP014881	2242951	2242235
specific region 9	APT_02060	-	hypothetical protein	AP014881	2243223	2242948
specific region 9	APT_02061	-	hypothetical protein	AP014881	2243717	2243304
specific region 9	APT_02062	-	phage major capsid protein HK97	AP014881	2245020	2243785
specific region 9	APT_02063	-	hypothetical protein	AP014881	2245533	2245087
specific region 9	APT_02064	-	hypothetical protein	AP014881	2245940	2245530

specific region 9-1	APT_02075	-	hypothetical protein	AP014881	2255163	2255002
specific region 9-1	APT_02076	-	hypothetical protein	AP014881	2255319	2255792
specific region 9-1	APT_02077	-	hypothetical protein	AP014881	2256244	2255753
specific region 10	APT_02152	-	hypothetical protein	AP014881	2330772	2330581
specific region 10	APT_02153	-	hypothetical protein	AP014881	2331749	2331111
specific region 10	APT_02154	-	hypothetical protein	AP014881	2332094	2331873
specific region 10	APT_02155	-	hypothetical protein	AP014881	2332755	2333351
specific region 10	APT_02156	*hsdM*	type I DNA methyltransferase M subunit HsdM	AP014881	2333387	2334913
specific region 10	APT_02157	*hsdS*	type I restriction-modification enzyme S subunit	AP014881	2334903	2336138
specific region 10	APT_02158	*hsdR*	type I site-specific deoxyribonuclease	AP014881	2336168	2339464
specific region 10	APT_02159	-	metal-dependent hydrolase	AP014881	2339467	2340204
specific region 10	APT_02160	-	hypothetical protein	AP014881	2340351	2341316
specific region 10	APT_02161	-	DEAD/DEAH box helicase	AP014881	2341313	2343952
specific region 10	APT_02162	-	hypothetical protein	AP014881	2344126	2344449
specific region 10	APT_02163	-	hypothetical protein	AP014881	2345307	2344672
specific region 10	APT_02164	-	plasmid stabilization protein	AP014881	2345605	2345856
specific region 10	APT_02165	-	plasmid stabilization protein	AP014881	2345853	2346158
specific region 10	APT_02166	-	hypothetical protein	AP014881	2346808	2346431

Table S3. List of experimentally validated mutations or insertion regions among two adapted strains, TH-3, and TI, of *A. pasteurianus* SKU1108.

Reference sequence	Mutated position in chromosome		Gene	Product	Mode of mutation or insertion		Locus_tag	Locus_tag (old)
GenBank acc. No.	TH-3	TI			TH-3	TI		
AP014881	-	84016	-	PAS/PAC sensor hybrid histidine kinase	-	H647Y	APT_00078	APT_0997
AP014881	190842	190925	*marR*	MarR family transcriptional regulator	R98H	Frameshift	APT_00170	APT_2081
AP014881	-	246307	-	hypothetical protein	-	1-bp insertion	Upstream of APT_00226	APT_1701
AP014881	248856	-	-	hypothetical protein	Frameshift	-	APT_00228	APT_1699
AP014881	250613	250748	*ansP*	L-asparagine permease	Frameshift	Transposon	APT_00229	APT_1698
AP014881	268640	-	-	D-alanyl-D-alanine serine-type carboxypeptidase	Q292L	-	APT_00250	APT_1083
AP014881	777821	-	-	hypothetical protein	6-bp deletion	-	APT_00732	APT_0215
AP014881	1385142	-	*asmA*	lipopolysaccharide biogenesis periplasmic protein AsmA	Silent mutation	-	APT_01316	APT_1527
AP014881	1565424	1565468	*dctA*	C4-dicarboxylate transporter	Transposon	Transposon	APT_01446	APT_2237
AP014881	1841392	-	-	hypothetical protein	G164S	-	APT_01702	APT_1971
AP014881	1909918	-	*glnD*	uridylyltransferase PII	6-bp deletion	-	APT_01763	APT_1249
AP014881	1935892	-	*mucR*	MucR family transcriptional regulator	4-bp deletion	-	APT_01785	APT_1226
AP014881	2000030	-	-	glycosyl transferase	Transposon	-	Upstream of APT_01848	APT_1213
AP014881	-	2266228	*cytC*	cytochrome c-552 class I	-	C39S	APT_02098	APT_2167

Table S4. List of homologous genes of 24 thermotoletant genes conserved in three *A. pasteurianus* genomes. The locus_tag of *A. tropicalis* SKU1100 strain is also shown.

gene	product	locus_tag			
SKU1108	SKU1108	SKU1108	NBRC 3283	386B	SKU1100
-	hypothetical protein	APT_00053	APA01_00570	APA386B_1540	ATPR_0586
hpnB	glycosyl transferase	APT_00180	APA01_01990	APA386B_1687	ATPR_1188
poxA	lysyl-tRNA synthetase	APT_00246	APA01_02750	APA386B_1758	ATPR_0143
lepA	GTP-binding protein LepA	APT_00584	APA01_06360	APA386B_2137	ATPR_0450
-	hypothetical protein	APT_00590	APA01_06420	APA386B_2143	ATPR_0443
mmpA	putative metalloprotease MmpA	APT_00603	APA01_06550	APA386B_2156	ATPR_0429
hsp20	heat shock protein Hsp20	APT_00623	APA01_06750	APA386B_2177	ATPR_3088
-	hypothetical protein	APT_00856	APA01_09110	APA386B_2415	ATPR_2096
glnE	glutamate-ammonia-ligase adenylyltransferase	APT_00857	APA01_09120	APA386B_2416	ATPR_2097
yhdJ	DNA methyltransferase	APT_00916	APA01_09690	APA386B_2479	ATPR_1965
minC	cell division inhibitor MinC	APT_00954	APA01_10070	APA386B_2523	ATPR_0029
-	hypothetical protein	APT_00961	APA01_10140	APA386B_2530	ATPR_0036
nhaK2	Na+/H+ antiporter	APT_00984	APA01_10370	APA386B_2553	ATPR_0071
smc	chromosome segregation protein SMC	APT_01022	APA01_10730	APA386B_2591	ATPR_1424
ttg2C	toluene ABC transporter periplasmic protein	APT_01083	APA01_11370	APA386B_2657	ATPR_0609
serA	D-3-phosphoglycerate dehydrogenase	APT_01136	APA01_11910	APA386B_2712	ATPR_0874
rpoE	RNA polymerase sigma-E factor (sigma-24) protein 2	APT_01242	APA01_12650	APA386B_100	ATPR_2218
xdhA	xanthine dehydrogenase XdhA	APT_01267	APA01_12900	APA386B_125	ATPR_2837
-	asparagine synthetase	APT_01828	APA01_17360	APA386B_665	ATPR_2801
-	flavodoxin/nitric oxide synthase	APT_01927	APA01_18620	APA386B_778	ATPR_1364
amiA	N-acetylmuramoyl-L-alanine amidase	APT_02041	APA01_19860	APA386B_897	ATPR_3151
cysG	siroheme synthase	APT_02252	APA01_21940	APA386B_1054	ATPR_0022
-	hypothetical protein	APT_02411	APA01_24150	APA386B_1217	ATPR_0162
htrA	endopeptidase DegP/Do	APT_02556	APA01_25660	APA386B_1373	ATPR_1619
